# Antioxidant Activity Enhancement Effect of Silver-Ionized Water: Silver Cation Prepared by Electrolysis

**DOI:** 10.3390/antiox12020467

**Published:** 2023-02-12

**Authors:** Tongjiao Wu, Santudprom Phacharapan, Natsuki Inoue, Yoshinori Kamitani

**Affiliations:** 1 The United Graduate School of Agricultural Sciences, Kagoshima University, 1-21-24 Korimoto, Kagoshima 890-0065, Japan; 2 Graduate School of Agricultural, Forestry and Fisheries, Kagoshima University, 1-21-24 Korimoto, Kagoshima 890-0065, Japan

**Keywords:** silver-ionized water, antioxidant activity, alkaline electrolyzed water, tourmaline water, near infrared (NIR) spectroscopy

## Abstract

In the present study, tap water, alkaline electrolyzed water (AlEW) and tourmaline water (TMW) were used as the electrolytes to generated the silver-ionized water (SIW), AlEW-SIW and TMW-SIW, respectively. The antioxidant properties of the samples containing ascorbic acid (AsA) were investigated by WST-kit method. The results showed that the SOD activity of AsA (2 mmol/L) dissolved in SIW (66.0%) was enhanced by about 8% compared to that of the tap water (57.9%). The SOD activity of the AlEW-SIW solution (77.3%), which was higher than that of the SIW solution, and lower than that of the AlEW solution (83.6%). The SOD activity of the TMW-SIW solution (83.0%) was similar to that of the TMW solution (82.5%). Furthermore, to classify the sample solutions, discriminant analyses were performed based on near infrared (NIR) spectral data, which was consistent with the results of the WST-kit method. The SOD activity of the AlEW-SIW and TMW-SIW solutions decreased slowly with storage time, and their SOD activities were higher than that of AlEW, TMW and the tap water solutions at storage time of 14 days. In summary, AlEW-SIW and TMW-SIW showed similar antioxidant activity enhancement as AlEW and TMW, and they also maintained the stability of the antioxidant activity of AsA during storage.

## 1. Introduction

Silver ions are used in a wide range of applications including the food industry, cosmetic processing and for medical treatment due to its antibacterial, antioxidant and anti-cancer properties [1,2,3,4,5,6]. Silver ions are generally generated by three methods such as chemical, nanotechnological and electrolytic [7,8,9]. Silver ions produced by electrolysis have been reported to have effective antibacterial properties [8]; however, there is scant research on the antioxidant properties. Currently, in the Japanese market a water has emerged as a drinking water with antioxidant properties called “silver ion cathode water”. Alkaline electrolyzed water (AlEW), which is also produced at a cathode, has been reported to show superoxide dismutase-like activity [10].

Hanaoka demonstrated that AlEW can enhance the superoxide dismutation (SOD) activity of antioxidants by electron spin resonance method [11]. Wu et al. demonstrated that two types of drinking water, AlEW and tourmaline water (TMW), could enhance the SOD activity of antioxidants in fruit juices by a simpler and faster method of SOD assay-kit water-soluble tetrazolium salt (WST) [12]. The WST-kit measures the scavenging ability of superoxide anions, while 2,2-diphenyl-1-picrylhydrazyl (DPPH) is mainly focused on the scavenging ability of free radicals. DPPH is sensitive to Lewis bases and interferes with quantitative analysis [13]. Therefore, the SOD activity of the sample solution can be analyzed more accurately by the SOD assay-kit WST method.

Near infrared (NIR) spectroscopy is a spectroscopic analysis method based on the absorption of NIR radiation by molecules. It is widely used to determine characteristics of various food products such as honey, coffee, fresh fruits and vegetables due to the low cost, rapidity of analysis and simple sample preparation [14,15,16]. However, little information is available on the analysis of drinking water by NIR spectroscopy.

In our previous report, it was demonstrated that TMW with micro-electric energy has the same effect as AlEW in enhancing the SOD activity of antioxidants [17]. Therefore, we considered whether silver-ionized water (SIW) generated by subjecting micro-electric currents to pure silver electrodes also has the effect of enhancing the activity of antioxidants. In the present study, the enhancement effect of silver-ionized water generated by electrolysis, a compound solution of SIW mixed with AlEW or TMW, and AlEW-SIW and TMW-SIW generated by applying microcurrent to silver electrodes with AlEW or TMW as electrolyte on antioxidant activity of ascorbic acid (AsA) was investigated for the first time. Moreover, the changes in antioxidant activity of SIW during storage were analyzed by the WST-kit method and NIR spectroscopy. The objective of the present study was to develop a new method to produce novel silver-ion drinking water with enhancement effect of antioxidant properties.

## 2. Materials and Methods

### 2.1. The Generation Principle of Electrolytic Silver-Ionized Water

Silver cations were prepared using electrolysis method through an electrolysis device which was developed by the Laboratory of Food and Environmental Systems Science, Kagoshima University (Kagoshima, Japan). In brief, two pure silver (length 50 mm, diameter 5 mm) electrodes with a spacing of 20 mm were placed in the electrolytic cell. The electrolyte is pumped into the electrolytic cell and the current and electrolyte flow rate are adjusted to obtain the corresponding concentration of silver-ionized water (the silver-ionized water generation device is shown in Figure S1 (Supplementary Materials)). Due to the extremely low electrical conductivity (EC) of pure water, minerals (e.g., NaHCO_3_, CaCO_3_ and MgCO_3_, etc.) are added to the pure water to increase the EC of the electrolyte during the electrolysis of silver, or tap water is used directly. By subjecting voltage to the electrodes, the oxidation reaction occurs at the anode and the reduction reaction at the cathode; the principle is as follows:
Positive pole (anode):2H_2_O → 4H^+^ + O_2_ + 4e^−^
Ag → Ag^+^ + e^−^Negative pole (cathode):2H_2_O + 2e^−^ → 2OH^−^ + H_2_

M^n+^ + ne^−^ → M
where “M^n+^” indicates the metal cation in the electrolyte and “M” means the metal atom recovered from the electrolyte on the cathode (negative pole).

### 2.2. Preparation of Test Water

Pure water was generated using a pure water generator (Elix Essential UV3; Merck Millipore, Germany). Alkaline Electrolyzed water (AlEW) was generated by an electrolyzed water generator (ROX-20TA; Hoshizaki Electric Co., Ltd., Aichi, Japan) at a current of 6, 8, 10 and 12 A, and a voltage of 10 V. Tourmaline water (TMW) was prepared with the tourmaline water generator (Kagoshima University self-developed experimental apparatus, Kagoshima Japan). A total of 120 g of Brazilian tourmaline stones were kept in a water bath to 25, 50, 75 and 100 °C for 30 min and then cooled down to room temperature. Tourmaline stones were placed in the tube of the TMW generator and 10 L of tap water was circulated by pump at a flow rate of 3.4 L/min for 30 min. Silver ionized water (SIW) was generated by electrolysis as described in Section 2.1, and the concentration of the produced SIW at different current values was measured by a silver ion meter (AGT-131; Japan Ion Co., Ltd., Tokyo, Japan). When sample solutions were prepared, the pH value (pH meter, TPX-999; Toko Chemical Laboratory Co., Ltd., Tokyo, Japan), the oxidation-reduction potential (ORP meter, TRX-90; Toko Chemical Laboratory Co., Ltd., Tokyo, Japan), electrical conductivity (electrical conductivity meter, CD-6021A; CUSTOM Co., Ltd., Tokyo, Japan), dissolved oxygen (dissolved oxygen meter, SG9-ELK; Mettler-Toledo Co., Ltd., Tokyo, Japan) and dissolved hydrogen (dissolved hydrogen meter, ENH-2000; TRUSTLEX Co., Ltd., Osaka, Japan) were measured, respectively. The physical and chemical parameters of sample solution used in the present study are shown in Table 1.

### 2.3. Determination of Superoxide Dismutation (SOD) Activity

SOD activity was measured by the SOD Assay Kit-WST method (Dojindo Molecular Technologies, Inc., Kumamoto, Japan). WST is a water-soluble tetrazolium salt. When superoxide radicals cause an oxidation reaction, WST-1 is reduced to WST-1 formazan and the color is changed to yellow; this reaction can be inhibited by SOD. Therefore, the activity of SOD can be calculated by colorimetric reaction. In order to study the antioxidant activity of each sample solution, AsA as a reducing agent with antioxidant properties was dissolved in the sample to prepare a sample solution with a concentration of 2 mmol/L. The details of the operation were performed according to our previous report [12].

### 2.4. Experimental Design and Treatments

In the present study, three experiments were designed to evaluate the antioxidant activity of each sample solution. In the first experiment, the effect of silver ion concentration on the antioxidant properties of SIW was verified. Tap water was used as the electrolyte and the flow rate was set to 1.0 L/min. The current was adjusted to obtain SIW with silver ion concentrations of 50, 100, 150 and 200 ppb. Tap water was used as control groups.

In the second experiment, the variation of antioxidant properties of SIW and AlEW mixed compound solution (called “Ag-AlEW compound solution” hereafter) and SIW produced by AlEW as an electrolyte (called “AlEW-SIW” hereafter) were evaluated. In brief, AlEW with an electrolytic current of 8 A (called “8A-AlEW” hereafter) was compounded with silver-ionized water at concentrations of 200 ppb at ratios of 0, 25, 50, 75 and 100%, thus analyzing the effect of the volume fraction of AlEW on the SOD activity of silver-ionized water. Next, 200 ppb of silver-ionized water was compounded with equal volume fractions (1:1) of AlEW with different electrolytic currents (6, 8, 10, 12 A) as well as a NaCl solution (as a control) with the same EC value as AlEW, SOD activity was measured; consequently, the effect of electrolytic strength of AlEW on the antioxidant activity of silver-ionized water was discussed. Then, 8A-AlEW instead of tap water was used as the electrolyte with a microcurrent of 2, 4, 6 and 8 mA to prepare AlEW-SIW and to analyze the changes of its antioxidant activity, and NaCl solution was used as a control group in this part of the experiment.

In the third experiment, the SOD activity of SIW and TMW mixed compound solution (called “Ag-TMW compound solution” hereafter) and SIW produced by TMW as an electrolyte (called “TMW-SIW” hereafter) was analyzed. Briefly, SOD activity was measured after compounding 200 ppb of SIW and TMW at different tourmaline stone treatment temperatures (25, 50, 75, 100 °C) as well as tap water (as control) in a 1:1 volume fraction. In addition, tap water was replaced by TMW (tourmaline stone treated at 75 °C) to prepare TMW-SIW, and its antioxidant properties were analyzed, where tap water was used as a control group.

### 2.5. Effect of Storage Time on the Antioxidant Activity of Each Sample Solution

According to the results of Section 2.4, sample solutions (10 mL) of AlEW, AlEW-SIW, TMW, TMW-SIW and tap water (as control) containing 2 mmol/L ascorbic acid were stored in a water bath at 25 °C under dark conditions. The antioxidant activity of each sample solution was measured at storage times of 0 h, 2 h, 4 h, 8 h, 24 h, 48 h, 3 d, 4 d, 5 d, 7 d and 14 d.

### 2.6. NIR Spectroscopy Analysis

NIR spectra were measured according to the report [18]. The NIR spectra (400–1100 nm) of samples were measured using a test tube (internal diameter: 17.6 mm) as a sample cell with the NIRS6500 in transmittance mode. The NIR measuring conditions were as follows: (1) the number of scans/spectrum: 50; (2) gain: ×1 and (3) wavelength: 400–1100 nm. A reference was measured using a blank test tube every five samples. Before NIR spectral measurement, the temperature of the test tube containing each sample solution was controlled at 25 °C using a water bath attached with a shaker. The NIR spectra of sample solutions were measured at storage times of 0 h, 4 h, 8 h, 4 d and 14 d. Pre-treatments such as smoothing, derivative and SNV were applied [19].

### 2.7. Statistical Analysis

Each measurement was repeated multiple times. The data from independent repeated experiments were obtained, and the means and standard deviations were calculated. All data were analyzed using Duncan’s multiple range test (SPSS17.0 for Windows; SPSS Inc., Chicago, IL, USA). Statistical significance was set at a value of *p* < 0.05. The Unscrambler software (CAMO Software AS, Oslo, Norway) and “R” software (free software) were employed to complete the discriminant analysis.

## 3. Results and Discussion

### 3.1. Effect of Silver ion Concentration on SOD Activity of SIW Solution

**Figure 1:**
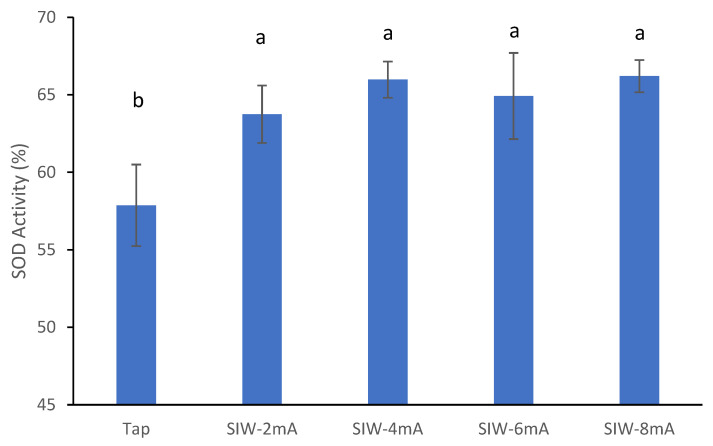
SOD activity of SIW solution with different concentrations. SIW-x mA, the electrolytic current of x mA for silver-ionized water (x = 2, 4, 6, 8). All sample solutions in this study contained 2 mmol/L AsA. Different letters indicate significant difference (*p* < 0.05). Each value is expressed as the mean value ± standard deviation of three replicates.

The effect of silver ion concentration on the antioxidant activity of SIW solution was investigated and the results are shown in Figure 1. Generally, the antioxidant activity of the SIW solution was significantly higher than that of the tap water solution (control). Specifically, the SOD activity of the SIW solution was 63.7, 66.0, 65.0 and 66.2% at electrolytic currents of 2, 4, 6 and 8 mA, respectively, which increased by about 8.1% compared to the control (57.9%). As shown in Table 1, the silver ion concentration of SIW was positively correlated with the electrolysis current; however, there was no significant difference in the SOD activity of SIW produced by various electrolysis currents. It can be demonstrated that the silver ion concentration is not the main factor affecting the SOD activity of SIW.

In addition, the SOD activity of SIW without added AsA was 0.09% (data not shown in the figure), showing almost no antioxidant properties. Hanaoka et al. reported that AlEW has no antioxidant properties by itself. When antioxidant substances were dissolved in AlEW, their dissociation was promoted and thus the antioxidant activity was enhanced [20]. It is supposed that the enhancement of antioxidant activity of AsA might be due to the promotion of AsA dissociation by SIW generated with microcurrent conditions. 

### 3.2. Antioxidant Activity of Silver Ion Compounded Solution

**Figure 2:**
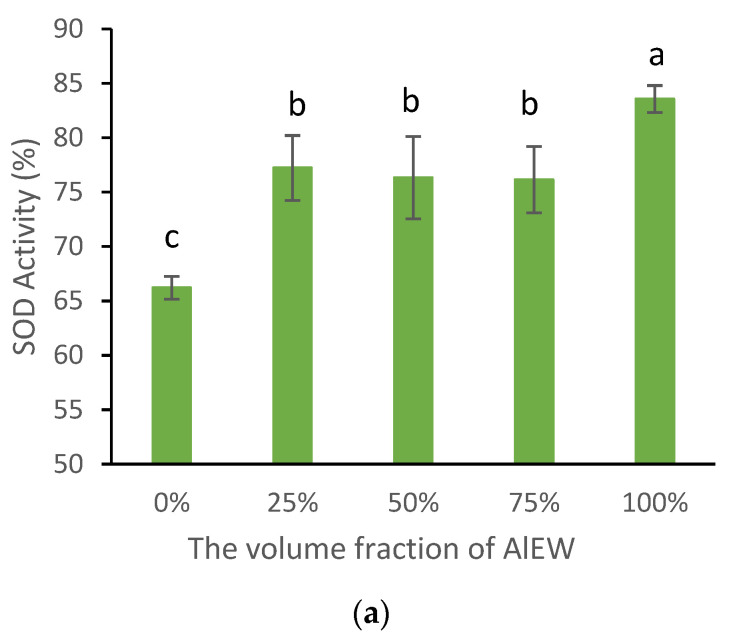

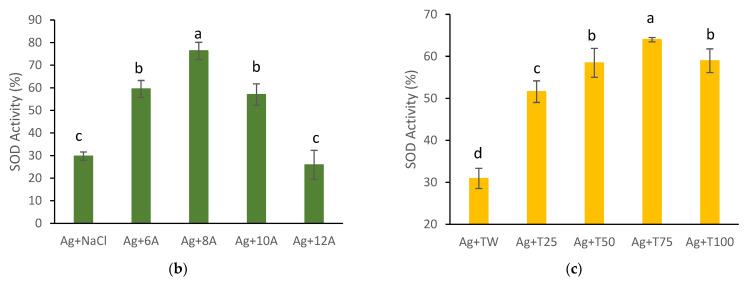
SOD activity of SIW compound solution; (**a**), The 200 ppb SIW compounded with different volume fraction of 8A-AlEW; (**b**), The 200 ppb SIW compounded in a 1:1 ratio with AlEW generated at different electrolytic currents; (**c**), The 200 ppb SIW compounded in a 1:1 ratio with TMW generated at different tourmaline stone treatment temperature. TW, tap water; Ag+6 A/T25, silver-ionized water is compounded with AlEW generated at an electrolytic current of 6 A or TMW generated at a tourmaline stone treatment temperature of 25 °C. Different letters indicate a significant difference (*p* < 0.05). Each value is expressed as the mean value ± standard deviation of three replicates. All sample solutions in this study contained 2 mmol/L AsA.

In our previous study, AlEW and TMW solutions were shown to be effective in enhancing the antioxidant activity of AsA, which was increased by 22.4% and 37.0% in AlEW and TMW, respectively, compared to that of AsA dissolved in pure water [17]. Moreover, in Section 3.1, it was concluded that the SOD activity of AsA was increased approximately 8% by the SIW solution. To further promote the effect of SIW in enhancing antioxidant activity, the SIW was compounded with AlEW and TMW in this section, and their antioxidant activities were investigated. The results are shown in Figure 2.

The SOD activity of SIW with silver ion concentration of 200 ppb and 8 A of AlEW compounded solution was greater than that of the SIW solution with 200 ppb (0%), indicating that the addition of AlEW enhanced the antioxidant activity of the Ag-AlEW compounded solution (Figure 2a). As the volume fraction of AlEW in the Ag-AlEW-compounded solution increased, the SIW was gradually diluted and the silver ion concentration decreased. However, the SOD activity of 25, 50 and 75% Ag-AlEW compounded solutions remained almost constant, which was in agreement with the Section 3.1.

**Figure 3:**
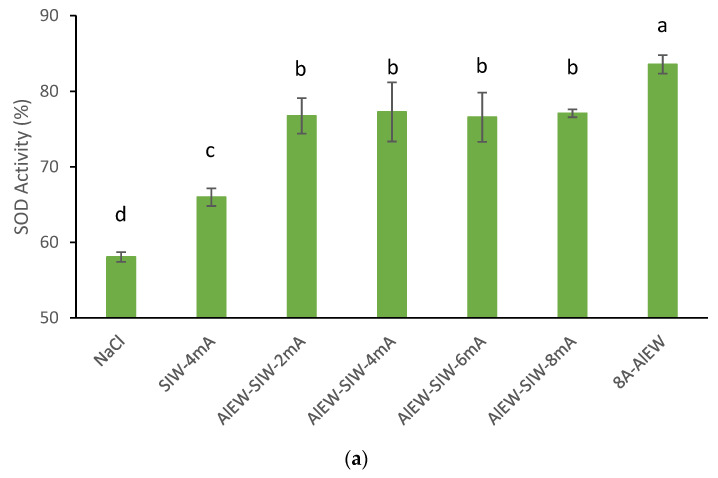

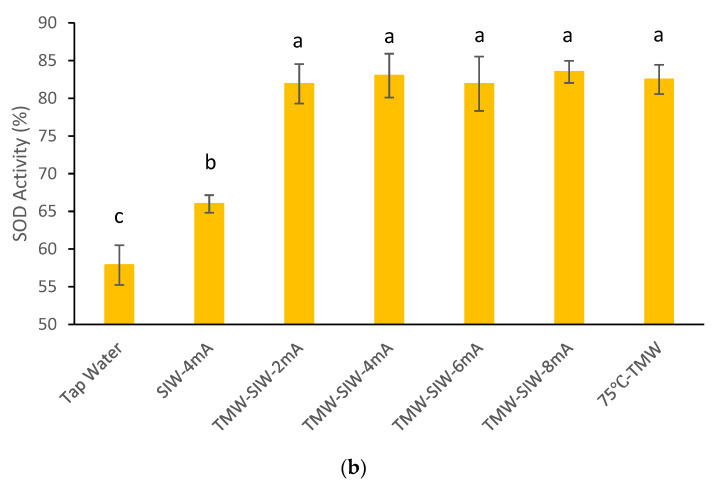
SOD activity of AlEW-SIW (**a**) and TMW-SIW (**b**) solutions. AlEW-SIW and TMW-SIW represent the silver-ionized water produced by using AlEW generated at an electrolytic current of 8 A or TMW generated at a tourmaline stone treatment temperature of 75 °C as the electrolyte, respectively. AlEW/TMW-SIW-x mA means the electrolytic current of x mA for silver-ionized water (x = 2, 4, 6, 8). NaCl solution and tap water were used as controls. Different letters indicate a significant difference (*p* < 0.05). Each value is expressed as the mean value ± standard deviation of three replicates. All sample solutions in this study contained 2 mmol/L AsA.

The antioxidant activity of 200 ppb SIW solution compounded with AlEW of different electrolytic currents was evaluated. As shown in Figure 2b, the SOD activity of the compounded solution showed an increasing tendency with the strengthening of the electrolytic current of AlEW. It reached a peak at 8 A and then gradually decreased, which was consistent with our previous report [17]. The SOD activity of the Ag-AlEW compounded solution was significantly higher than that of the control except for the electrical current of 12 A (*p* < 0.05). Similar results were obtained when SIW was compounded with TMW with different tourmaline stone treatment temperatures (Figure 2c). The SOD activity of Ag-TMW compounded solution showed a trend of increasing and then decreasing, and obtained a maximum at 75 °C, and was significantly higher than that of the control (SIW compounded with tap water). Moreover, the SOD activities of Ag-AlEW and Ag-TMW compounded solutions were significantly lower than those of 8A-AlEW (83.6% as shown in Figure 3a) and 75 °C-TMW (82.5% as shown in Figure 3b) at the peak. There are two potential causes for this phenomenon. First, since the electrolyte used to generate AlEW is NaCl, when mixed with SIW, Cl^−^ and Ag^+^ react to form AgCl precipitate, which affects the SOD activity of the compounded solution. However, the raw material used to produce TMW is tap water and its Cl^−^ content is much less than that of AlEW. The second reason may be the dilution of AlEW or TMW by mixing with SIW which resulted in a reduction in the enhancement of antioxidant activity. Therefore, further research is necessary to determine the cause of the phenomenon.

### 3.3. Antioxidant Activity of AlEW-SIW and TMW-SIW Solutions

To eliminate the effect of dilution of AlEW or TMW mixed with SIW resulting in low antioxidant activity enhancement, the electrolyte (tap water) used to produce SIW was replaced by 8A-AlEW or 75 °C-TMW with the peak obtained in Section 3.2. The antioxidant activities of the generated AlEW-SIW and TMW-SIW solutions were analyzed, and the results are shown in Figure 3.

The SOD activity of the AlEW-SIW solution (77.3%) was significantly higher than that of the SIW-4mA solution (66.0%) and the NaCl solution (58.1%), however, with the increase in the electrolytic current, the SOD activity of AlEW-SIW solution remained basically constant and significantly lower than the 8A-AlEW solution of which the SOD activity was 83.6% (Figure 3a). Moreover, the silver ion concentration of AlEW-SIW was always maintained at a low concentration level of about 10 ppb despite the change of electrolytic current (Table 1). Admittedly, silver ions are relatively active and react with Cl^−^ to form AgCl precipitates [21] resulting in low silver ion concentration in AlEW-SIW, which affects the antioxidant activity of the AlEW-SIW solution. In addition, AlEW has a favorable antioxidant activity enhancing effect that has been reported [22,23], and it can be inferred that the reason why AlEW-SIW can enhance the antioxidant activity of AsA may be the main role performed by AlEW.

The SOD activity of the TMW-SIW solution (Figure 3b) obtained similar results to the AlEW-SIW solution. The contrast is that the SOD activity of the TMW-SIW solution (83.0%) is maintained at the same level as that of the TMW solution (82.5%). This confirmed the previous inference that the SOD activity of Ag-TMW compounded solution was less than that of the 75 °C TMW solution due to the dilution of TMW by mixing with SIW. This indicated that AlEW or TMW as an electrolyte contributed to the enhanced antioxidant activity of AsA by silver-ionized water.

### 3.4. Effect of Storage Time on the Antioxidant Activity of SIW Solution

**Figure 4:**
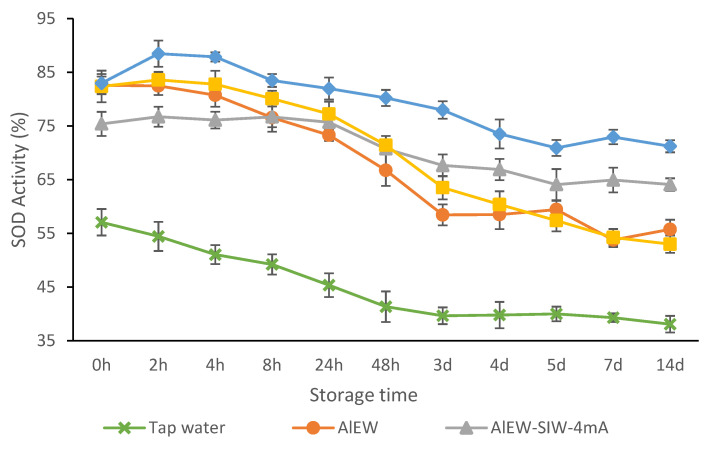
The effect of storage time on the antioxidant activity of each sample solution. AlEW, alkaline electrolyzed water; TMW, tourmaline water; AlEW/TMW-SIW-4mA, silver-ionized water produced by using AlEW or TMW as electrolyte at 4 mA; Tap water was used as control. Each value is expressed as the mean value ± standard deviation of three replicates. All sample solutions in this study contained 2 mmol/L AsA.

In Section 3.3, we discussed the antioxidant activity of the SIW solution prepared with functional water with antioxidant enhancement effect as electrolyte and concluded that the TMW-SIW solution had the same enhancement effect on AsA antioxidant activity as the 75 °C TMW solution. Since there was no significant difference between the SOD activities of the TMW-SIW solution with various silver ion concentrations, to comply with the WHO regulations on the silver ion content in drinking water disinfection [24], the antioxidant activity of the TMW-SIW solution with a silver ion concentration of 100 ppb (electrolytic current of 4 mA) was measured in this section. To maintain the identical experimental conditions, the electrical current value of AlEW-SIW was same as that of TMW-SIW. The effect of storage time on the SOD activity of AlEW, AlEW-SIW-4mA, TMW, TMW-SIW-4mA and tap water (as control) solutions was evaluated and the results are shown in Figure 4. In general, the SOD activity of each sample solution showed a decreasing tendency with the storage time, and the SOD activities of the AlEW, AlEW-SIW-4mA, TMW and TMW-SIW-4mA solutions were significantly higher than those of the control group during storage (*p* < 0.05).

AlEW and TMW solutions had similar trends of SOD activity. During the storage time from 0 h to 4 h, the SOD activity of the AlEW and TMW solutions remained basically stable, and from 4 h the SOD activity decreased rapidly. At the storage time from 3 d to14 d, the SOD activity of the TMW solution still showed a decreasing trend, and the rate of decrease was lower compared with that from 0 d to 3 d, while the SOD activity of the AlEW solution decreased but basically remained gentle. For the control group, the SOD activity decreased rapidly during the storage time from 0 d to 3 d, and remained basically stable at the storage time from 3 d to 14 d.

The SOD activity of AlEW-SIW solution stabilized from 0 h to 24 h and showed a decreasing tendency from 24 h until 5 d when it tended to level off again. At 0 h, the SOD activity of the AlEW-SIW solution was significantly lower than that of the AlEW solution, however, it was equal to that of the AlEW solution at 8 h and significantly higher than that of the AlEW solution from 48 h. For the TMW-SIW solution, the SOD activity increased slightly at the storage time from 0 h to 2 h, then decreased gradually at the storage time from 4 h to 5 d, and levelled off subsequently. At the storage time of 48 h, the SOD activity was lower than that at the 0 h. Furthermore, the SOD activity at 0 h was equal to that of the TMW solution; from the storage time of 2 h, its SOD activity surpassed that of the TMW solution, and during the following storage period, the SOD activity of the TMW-SIW solution was significantly higher than that of the TMW solution (*p* < 0.05).

AsA is an extremely oxidizable antioxidant that reacts with silver ions in solution to form dehydroascorbic acid (DHAsA). Generally in the organism, AsA and DHAsA can be interconverted by redox reactions and both of them possess bioactivities [25,26]. AsA was converted to DHAsA in the presence of metal ions, and DHAsA dissolved in functional water was reduced to AsA again by AlEW and TMW, which are functional waters with reducing properties. Therefore, it is hypothesized that this might be one of the reasons for the effect of maintaining the overall antioxidant activity of the solution. However, the specific mechanisms that influence SIW to enhance the antioxidant activity of AsA are multifaceted and need further investigation.

In conclusion, compared with AlEW and TMW, AlEW-SIW and TMW-SIW generated with them as electrolytes can then more effectively maintain AsA with a higher antioxidant activity and reduce the rate of decrease in their SOD activity.

### 3.5. NIR Spectroscopy Analysis

**Figure 5:**
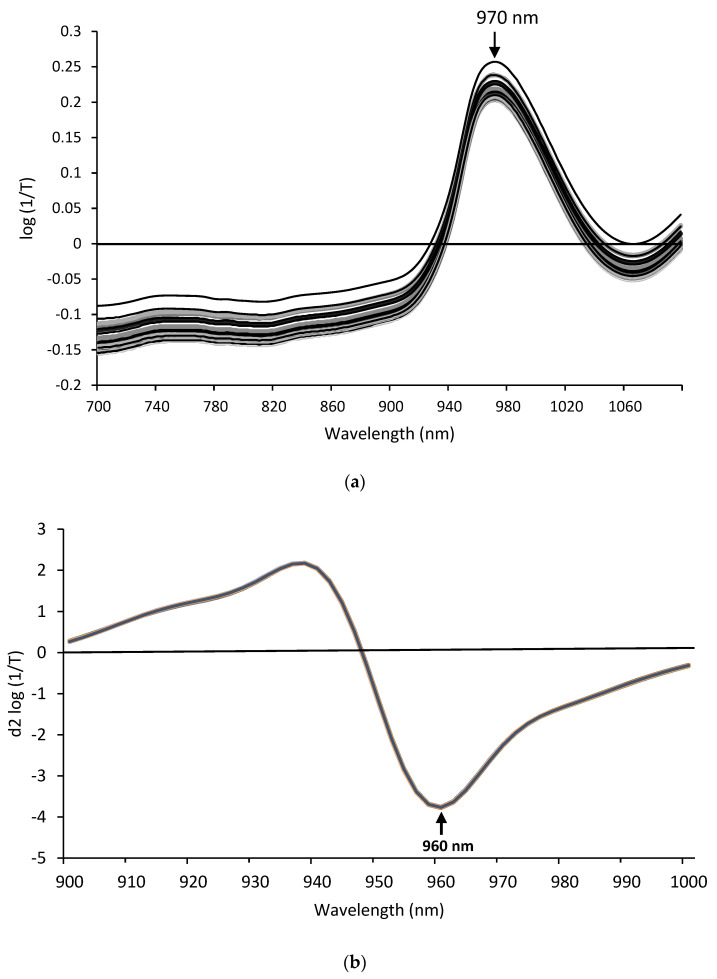
The NIR spectra of 5 types of sample solutions at 0 h–14 days. (**a**), original spectra; (**b**), second derivative spectra used for discriminant analysis. Different color lines indicate each NIR spectra of different sample solutions.

To classify the sample solutions with a different storage time, discriminant analyses were performed based on NIR spectral data. The NIR spectra of sample solutions are shown in Figure 5. The water band was observed at 970 nm in Figure 5a. Smoothing and second derivative treatments using the Savitsky-Golay method, and SNV treatment were performed to diminish spectral noise and correct baseline shifts. The SNV-treated second derivative spectra from 900 nm to 1000 nm used for the discriminant analysis are shown in Figure 5b. To perform the discriminant analyses, 17 variables from d^2^ log (1/T) value at 900 nm to that at 996 nm were used.

**Figure 6:**
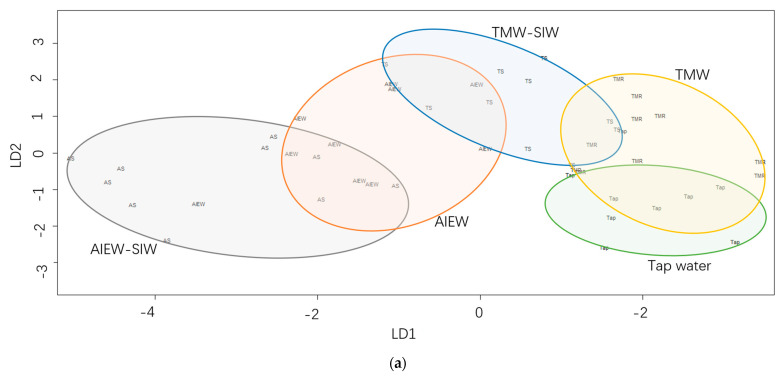

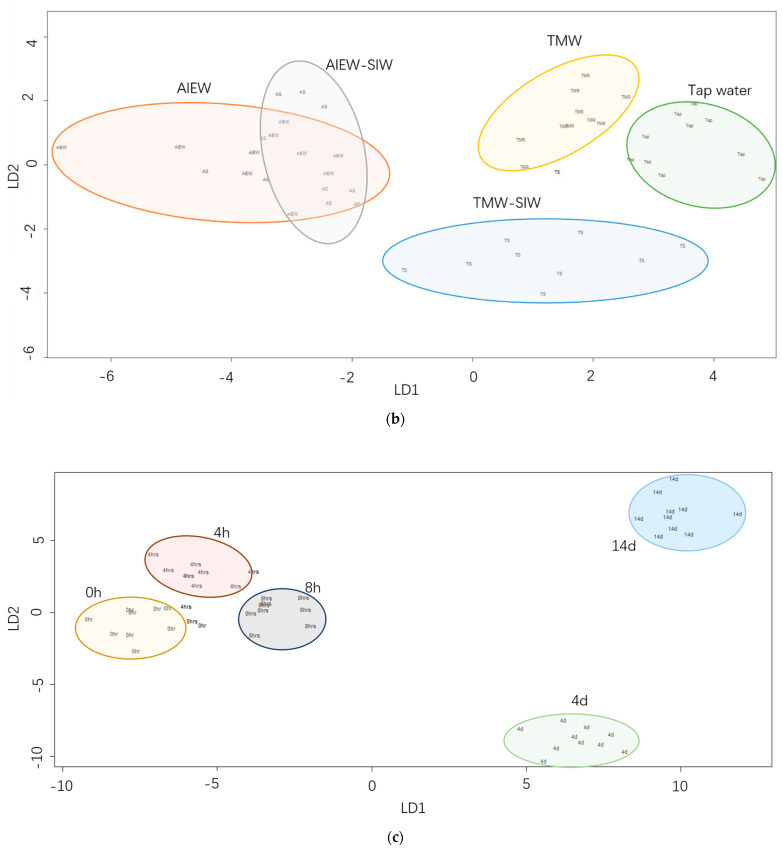

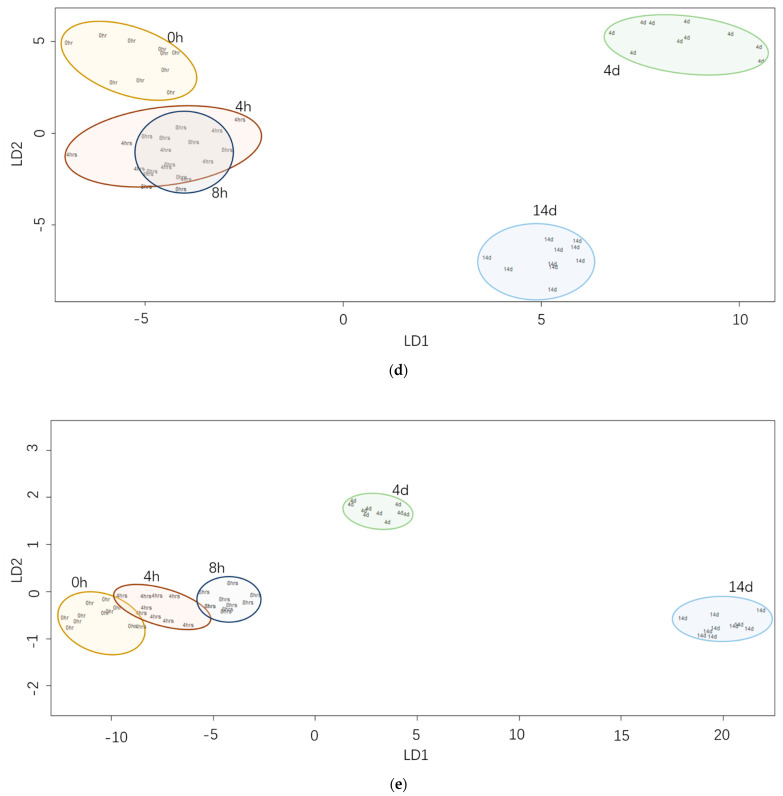
Score plots of discriminant analysis. (**a**,**b**), discriminant score plots for the five sample solutions at 0 h and 14 d of storage, respectively. (**c**–**e**), discriminant score plots for tap water, TMW-SIE and AlEW-SIW solutions during storage (0 h–14 d), respectively. AlEW, alkaline electrolyzed water; TMW, tourmaline water; AlEW/TMW-SIW-4mA, silver-ionized water produced by using AlEW or TMW as electrolyte at 4 mA. All sample solutions in this study contained 2 mmol/L AsA.

Each sample solution was subjected to discriminant analyses and mapped onto a plane to confirm identity by the area, density and distance indicated by each sample cluster. The plots of the discriminant scores are shown in Figure 6. The clusters of the tap water, TMW and TMW-SIW solutions overlapped with each other, while the cluster of the AlEW-SIW solution was separated from the other clusters except for the AlEW solution. Similarly, the cluster of the tap water solution was overlapped with the cluster of the TMW solution but separated from the other three clusters (Figure 6a). In contrast, the distribution of discriminant score plots for the storage time of 14 d had a notable change as shown in Figure 6b. The clusters of the tap water, TMW and TMW-SIW solutions were separated from each other except for the clusters of the AlEW and AlEW-SIW solutions. As shown in Table 1, the TMW and tap water solutions had similar pH and EC values because TMW was produced by flowing tap water through tourmaline stones. Likewise, AlEW-SIW was generated by subjecting AlEW to microcurrent and their solutions had similar pH, EC and DO values (Table 1). Therefore, it was inferred that the overlap between the clusters of TMW and tap water solutions (Figure 6a) as well as the overlap between AlEW-SIW and AlEW groups (Figure 6a,b) might be due to their similar physicochemical properties.

As shown in Figure 6d,e, the plots of discriminant scores of samples stored for 0 h, 4 h and 8 h were distributed in virtually the same area as in the plane of LD1–LD2 in the case of TMW-SIW and AlEW-SIW solutions. This corresponded to the result that the SOD activities of TMW-SIW and AlEW-SIW solutions were nearly identical during this storage time, as shown in Figure 4. In the case of tap water solution, the plots of discriminant scores of samples stored for 0 h, 4 h and 8 h were dispersed in the plane of LD1–LD2 as shown in Figure 6c. This was in line with the result that the SOD activities of tap water solution gradually decreased during this storage time, as shown in Figure 4.

The plots of discriminant scores of the TMW-SIW, AlEW-SIW and tap water solutions stored for 4 d and 14 d, on the other hand, were entirely separated in the plane of LD1–LD2, as shown in Figure 6. As shown in Figure 4, this was consistent with the results that the SOD activities of the aforementioned three types of sample solutions decreased during this storage time.

From these results mentioned above, it was suggested that the NIR spectra of each sample solution containing AsA (2 mmol/L) reflected information on the SOD activities. In order to fully understand the relationship between the NIR spectra and the antioxidant activity of functional water containing AsA, further study is needed.

## 4. Conclusions

The antioxidant activity of the SIW, Ag-AlEW or Ag-TMW compounded solutions and the AlEW-SIW or TMW-SIW solutions was investigated. The results showed that SIW enhanced the SOD activity of AsA. Compounding with AlEW or TMW contributed to the enhanced antioxidant activity of SIW solution, while the SOD activity of the compounded solution was significantly lower than that of the AlEW and TMW solutions. The results also showed that the antioxidant activity of the solutions of AlEW-SIW and TMW-SIW generated by AlEW or TMW as electrolyte was significantly higher than that of the SIW solution. However, the SOD activity of the AlEW-SIW solution was significantly lower than that of the AlEW solution, while TMW-SIW had the same enhancement effect as TMW. The SOD activity of the AlEW, TMW and tap water (control) solutions decreased rapidly with storage time, while that of the AlEW-SIW-4mA and TMW-SIW-4mA solutions maintained as constant at the initial stage of storage time (0–24 h), although there was a slight decrease during storage, and it was significantly higher than that of the AlEW, TMW and control solutions at the final stage of storage time. Moreover, the differences in the sample solutions at each storage time can be classified by discriminant analyses of the NIR spectra, which are in agreement with the results of the WST-kit method. In conclusion, AlEW-SIW and TMW-SIW showed similar antioxidant activity enhancement as AlEW and TMW, and they also maintained the stability of the antioxidant activity of AsA during storage.

## Figures and Tables

**Table 1:** Physical and chemical parameters of sample solution used in the present study *.

Sample Solution	C-Ag^+^ (ppb)	pH	EC (μS/cm)	DO (mg/L)	ORP (mV)	DH (ppm)
Tap Water	-	7.66 ± 0.04 gh	142.2 ± 1.8 f	7.40 ± 0.14 bc	234.0 ± 15.5 f	-
NaCl	-	6.92 ± 0.03 i	1001.5 ± 1.3 d	7.00 ± 0.10 def	168.0 ± 2.6 g	-
SIW-2 mA	40	7.84 ± 0.19 ef	145.2 ± 0.7 f	7.15 ± 0.35 cde	231.5 ± 0.7 f	-
SIW-4 mA	100	7.77 ± 0.26 efg	144.6 ± 1.3 f	7.35 ± 0.07 bcd	232.0 ± 1.4 f	-
SIW-6 mA	150	7.13 ± 0.28 fgh	146.6 ± 0.8 f	7.43 ± 0.50 bc	243.3 ± 6.4 e	-
SIW-8 mA	210	7.85 ± 0.18 e	149.7 ± 0.2 f	7.15 ± 0.49 cde	236.0 ± 0.0 ef	-
AlEW6 A	-	9.63 ± 0.01 d	853.2 ± 5.3 e	5.16 ± 0.71 bcd	−110.7 ± 7.2 h	0.23 ± 0.01 g
AlEW8 A	-	10.26 ± 0.00 c	998.8 ± 4.1 d	7.35 ± 0.21 l	−215.5 ± 3.5 m	0.39 ± 0.02 c
AlEW10 A	-	10.44 ± 0.00 b	1397.0 ± 2.4 b	8.06 ± 0.42 a	−174.5 ± 6.3 k	0.43 ± 0.00 b
AlEW12 A	-	10.93 ± 0.02 a	1721.5 ± 2.2 a	7.15 ± 0.14 cde	−198.3 ± 4.2 l	0.48 ± 0.01 a
AlEW-SIW-2 mA	10	10.35 ± 0.01 bc	1023.5 ± 6.2 c	7.65 ± 0.35 b	−138.5 ± 3.5 j	0.30 ± 0.00 e
AlEW-SIW-4 mA	20	10.34 ± 0.00 bc	1009.0 ± 1.1 d	7.40 ± 0.28 bc	−122.0 ± 1.4 i	0.27 ± 0.02 f
AlEW-SIW-6 mA	10	10.35 ± 0.02 bc	1027.5 ± 2.2 c	7.00 ± 0.42 def	−129.5 ± 7.7 ij	0.27 ± 0.05 f
AlEW-SIW-8 mA	10	10.34 ± 0.00 bc	1030.5 ± 2.7 c	6.95 ± 0.07 efg	-137.5 ± 1.9 j	0.32 ± 0.02 d
TMW25 °C	-	7.56 ± 0.04 h	139.1 ± 6.1 f	6.62 ± 0.87 ghi	310.7 ± 3.5 d	-
TMW50 °C	-	7.77 ± 0.09 efg	136.6 ± 5.8 f	6.20 ± 0.14 jk	349.5 ± 2.8 a	-
TMW75 °C	-	7.68 ± 0.01 fgh	135.6 ± 4.6 f	6.75 ± 0.21 fgh	328.8 ± 1.7 c	-
TMW100 °C	-	7.74 ± 0.01 efg	137.8 ± 2.8 f	6.82 ± 0.17 fgh	316.6 ± 2.1 d	-
TMW-SIW-2 mA	60	7.66 ± 0.03 gh	136.4 ± 1.6 f	6.10 ± 0.28 k	329.5 ± 9.1 c	-
TMW-SIW-4 mA	100	7.69 ± 0.06 fgh	140.1 ± 0.4 f	6.75 ± 0.07 fgh	339.0 ± 8.5 b	-
TMW-SIW-6 mA	150	7.70 ± 0.03 fgh	138.7 ± 1.0 f	6.60 ± 0.15 hi	337.0 ± 4.2 bc	-
TMW-SIW-8 mA	220	7.68 ± 0.02 fgh	141.6 ± 1.3 f	6.50 ± 0.00 ij	339.5 ± 3.3 b	-

* Tables may have a footer. 1 Tap, tap water; SIW-x mA, the electrolytic current of x mA for silver-ionized water (x = 2, 4, 6, 8); AlEW, alkaline-electrolyzed water; TMW, tourmaline water; AlEW-SIW/TMW-SIW, silver-ionized water generated with AlEW/TMW as electrolyte; C-Ag^+^, the concentration of silver ion; EC, electrical conductivity; DO, dissolved oxygen; ORP, oxidation-reduction potential; DH, dissolved hydrogen. “-” means not detected. Different letters indicate significant difference (*p* < 0.05). Each value is expressed as the mean value ± standard deviation of three replicates. All sample solutions in this study contained 2 mmol/L AsA.

## Data Availability

Data is contained within the article or supplementary material.

## Supplementary Materials

The following supporting information can be downloaded at: https://www.mdpi.com/article/10.3390/antiox12020467/s1, Figure S1: The silver ionized water generation device.

## Abbreviations

AlEW	Alkaline electrolyzed water
TMW	Tourmaline water
SIW	Silver-ionized water
AlEW-SIW	Silver-ionized water produced by using AlEW
TMW-SIW	Silver-ionized water produced by using TMW
AsA	Ascorbic acid
SOD	Superoxide dismutation
WST	Water-soluble tetrazolium salt
DPPH	2,2-diphenyl-1-picrylhydrazyl
NIR	Near infrared
EC	Electrical conductivity
ORP	Oxidation-reduction potential
DO	Dissolved oxygen
DH	Dissolved hydrogen
DHAsA	Dehydroascorbic acid
