# Supplementary figures and images for: Antioxidant Activity Enhancement Effect of Silver-Ionized Water: Silver Cation Prepared by Electrolysis
Source: Antioxidants (Basel). 2023 Feb 12;12(2):467. doi: 10.3390/antiox12020467 (PMC9952159; doi:10.3390/antiox12020467)

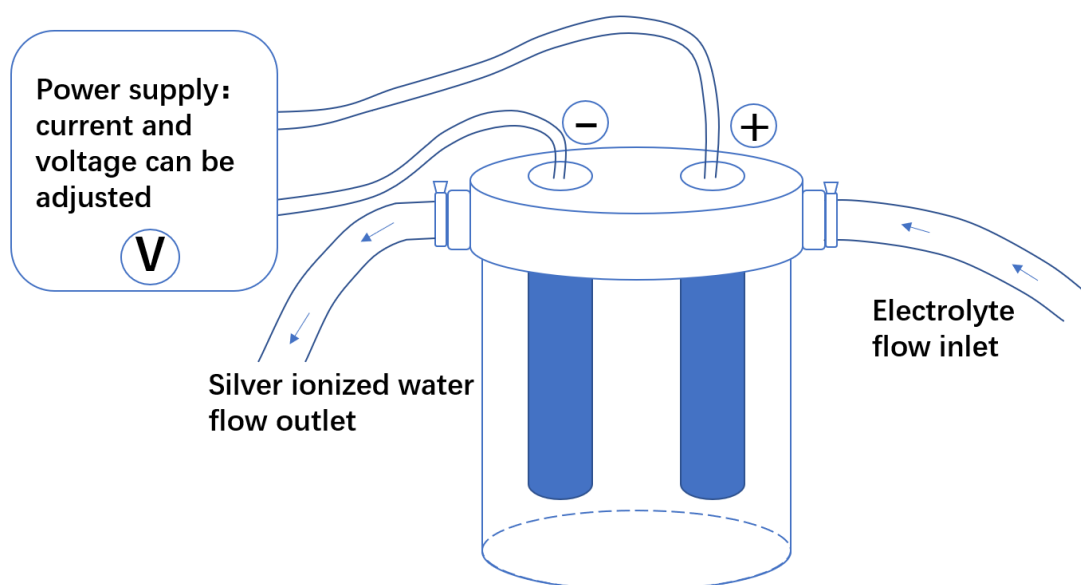

**Figure S1.** The silver ionized water generation device

Supplement: Supplementary file 1 [file antioxidants-12-00467-s001.zip › antioxidants-2188955-supplementary.pdf]
